# Systemic Medications and Their Ocular Side Effects

**DOI:** 10.7759/cureus.74976

**Published:** 2024-12-02

**Authors:** Mashael Al-Namaeh

**Affiliations:** 1 Department of Clinical Research, Clinical Virtual Research Center, Wayne, USA; 2 Department of Optometry, Kentucky College of Optometry, Pikeville, USA

**Keywords:** antituberculous ocular toxicity, ocular toxicity, retinal toxicity, systemic medication, tamoxifen ocular toxicity, visual field defect

## Abstract

Most of the drugs that we use in our everyday clinic cause ocular side effects or toxicity, depending on the drug duration and dose. Eye care physicians should be familiar with any possible ocular side effects linked to these medications, which could save the physicians' time to determine the diagnosis of the ocular irritation or toxicity. Not all medications are listed in this review, but we did go over the most common systemic medications based on our experience seeing patients in our everyday clinic. The ocular adverse effects of the most common drugs used are reviewed in this review article.

## Introduction and background

In our everyday practice, we observe that many systemic medications our patients are taking have adverse effects that affect the eyes. Eye care physicians should be familiar with any possible ocular side effects linked to these medications. An update on the ocular side effects of the most widely prescribed drugs for medical conditions is given in this paper. All systemic medication ocular side effects covered in this review article are summarized in Table 1, categorized as anterior segment, posterior segment, neurological, and others.

**Table 1:** Ocular side effects associated with the most common therapeutics usage categorized by anterior segment, posterior segment, and neurological signs and symptoms HTLV-1: human T-lymphotropic virus type 1, IOP: intraocular pressure, SSRIs: selective serotonin reuptake inhibitors, ACE: angiotensin-converting enzyme, CMV: cytomegalovirus, SGLT2: sodium-glucose cotransporter 2

Drug class	Drug generic (brand name) or drug class		Potential ocular side effect (anterior segment)
Antiarrhythmic drugs	Amiodarone (Pacerone)		Corneal deposits, cornea verticillata (vortex keratopathy, whorl keratopathy, Fleischer vortex)
Antibiotics: aminoglycosides	Gentamicin (Garamycin)		Rubeosis iridis, neovascular granuloma
Antibiotics: sulfonamides	Sulfonamides		Conjunctival scarring
Antibiotics: synthetic penicillin	Synthetic penicillin		Dry eye syndrome, mild eye redness, itching
Antibiotics: tetracyclines	Tetracycline (Sumycin) or minocycline (Minocin)		Conjunctival and sclera pigmentation (irreversible)
Antibody-drug conjugates: anti-tumor necrosis factor-alpha inhibitors	Infliximab		Bilateral rosacea-like keratopathy
Antibody-drug conjugates: interleukin-6 receptor inhibitors	Tocilizumab		Viral conjunctivitis, ophthalmic herpes zoster infection, HTLV-1 uveitis
Anticancer drugs: immune checkpoint inhibitors	Pembrolizumab and nivolumab		Dry eye syndrome, blepharitis, conjunctivitis, uveitis, scleritis
Anticancer drugs: tyrosine kinase inhibitors	Imatinib and ripretinib		Epiphora, periorbital edema, subconjunctival hemorrhages
Anticancer drugs: cytotoxic agents	Cisplatin		Blepharitis, conjunctivitis, eyelid swelling
Anticancer drugs: immune-based cancer drugs	Rituximab		Transient IOP elevations, iridocyclitis, conjunctivitis, periorbital edema
Antidepressants	Amitriptyline (Elavil) and SSRIs		Dry eye syndrome, dilated pupils, cataract, difficulty lens accommodation, acute closed-angle glaucoma, elevated IOP
Antiepileptic drugs	Topiramate (Topamax)		Increased pressure, acute angle-closure glaucoma
Antihypertensive: ACE inhibitors	Lisinopril		Photophobia, conjunctivitis
Antihypertensive: beta-blockers	Metoprolol		Dry eye syndrome, conjunctivitis, corneal epithelial damage, lower IOP
Antihypertensive: calcium channel blockers	Amlodipine		Open-angle glaucoma, normal tension glaucoma
Antihistamines	Antihistamines		Dry eye syndrome, keratitis sicca, mydriasis, decreased accommodation, anisocoria
Antipsychotics: phenothiazines	Thioridazine (Mellaril) and chlorpromazine (Thorazine)		Pigmentary cornea, abnormal eyelid pigmentation, intrapalpebral conjunctival pigmentation, anterior lens capsule pigmentation, anterior and posterior subcapsular cataracts
Antipsychotics: atypical	Olanzapine		Acute angle-closure glaucoma, periorbital edema
Antipsychotics: mood stabilizers	Lithium		Dry eye syndrome
Antitubercular drugs	Rifamycin (Aemcolo)		Hypopyon, anterior uveitis, intermediate uveitis, tears stain the contacts
Antiviral	Cidofovir		Anterior uveitis
Antiviral	Ganciclovir		Conjunctivitis, dry eye syndrome, cataract
Bisphosphonates	Alendronate, risedronate, ibandronate		Conjunctivitis, eyelid edema, periorbital edema, uveitis, scleritis
Dermatologic agents	Isotretinoin (Accutane)		Corneal opacities, keratitis, dry eye, meibomian gland dysfunction, blepharoconjunctivitis
Hormones	Levothyroxine (Synthroid)		Eyelid hyperemia
Non-steroidal anti-inflammatory drug	Ibuprofen (Advil)		Dry eye syndrome, xerosis (dryness)
Selective alpha 1 blockers	Tamsulosin (Flomax)		Intraoperative floppy iris syndrome
Steroids	Prednisolone (Pediapred)		Cataract (anterior subcapsular lens), steroid-induced glaucoma
Drug class	Drug generic (brand name) or dug class		Potential ocular side effect (posterior segment)
Antiepileptic drugs	Phenytoin		Acute bilateral vision loss
Antibiotics: aminoglycosides	Gentamicin (Garamycin)		Retinal hemorrhages, retinal edema, cotton-wool spots, arteriolar narrowing, venous beading, pigmentary retinopathy, optic atrophy
Antibiotics: nalidixic acid	Nalidixic acid (Nevigramon)		Swollen optic nerves, papilledema
Antibiotics: fluoroquinolones	Fluoroquinolones		Retinal detachment
Antibiotics: tetracyclines	Tetracycline (Sumycin) or minocycline (Minocin)		Macula hyperpigmentation (irreversible), idiopathic intracranial hypertension
Antibody-drug conjugates: anti-tumor necrosis factor-alpha inhibitors	Infliximab		Retinal vein thrombosis, optic neuritis, CMV retinitis, toxoplasmic chorioretinitis
Antibody-drug conjugates: interleukin-6 receptor inhibitors	Tocilizumab		Fulminant bilateral papilledema
Anticancer drugs: cytotoxic agents	Cisplatin		Retinal ischemia, retinal toxicity
Anticancer drugs: immune-based cancer drugs	Rituximab		Retinal necrosis, macular edema, CMV retinitis
Anti-diabetic SGLT2 inhibitors	Canagliflozin		Vitreous hemorrhages
Anti-diabetic GLP-1 agonists	Semaglutide		Non-arteritic anterior ischemic optic neuropathy
Anti-estrogen	Tamoxifen (Nolvadex)		Crystalline maculopathy
Antihypertensive ACE inhibitors	Lisinopril		Retinal hemorrhages
Antimalarial drugs	Hydroxychloroquine (Plaquenil) and chloroquine (Aralen)		Bull's eye maculopathy
Antipsychotics: phenothiazines	Thioridazine (Mellaril) and chlorpromazine (Thorazine)		Pigmentary retinopathy, salt and pepper fundus
Antipsychotics: atypical	Olanzapine		Retinal vein occlusion
Antitubercular drugs	Rifampin (Rifadin)		Panuveitis, retinal vasculitis
Antitubercular drugs	Ethambutol (Myambutol)		Optic neuritis (retrobulbar)
Antiviral	Ganciclovir		Retinal detachment, macular edema, vitreous floaters
Bisphosphonates	Alendronate, risedronate, ibandronate		Optic or retrobulbar neuritis
Dermatologic agents	Isotretinoin (Accutane)		Idiopathic intracranial hypertension
Hormones	Levothyroxine (Synthroid)		Idiopathic intracranial hypertension
Non-steroidal anti-inflammatory drug	Ibuprofen (Advil)		Optic neuritis
Other	Elmiron (pentosan polysulfate sodium)		Pigmented maculopathy
Drug class		Drug generic (brand name) or drug class	Potential ocular side effect (neurological)
Antibiotic		Nalidixic acid (Nevigramon)	Headaches, color vision defects, reversible sixth nerve palsy
Antipsychotics: phenothiazines		Thioridazine (Mellaril) and chlorpromazine (Thorazine)	Nyctalopia, brown vision
Antipsychotics: atypical		Olanzapine	Oculogyric crisis
Antipsychotics: mood stabilizers		Lithium	Skew deviation, downbeat nystagmus
Antitubercular drugs		Phenytoin (Dilantin)	Nystagmus, diplopia
Anticancer drugs: immune checkpoint inhibitors		Pembrolizumab and nivolumab	Myasthenia gravis
Dermatologic agents		Isotretinoin (Accutane)	Ocular pseudo-myasthenic reaction
Hormones		Levothyroxine (Synthroid)	Visual hallucinations
Non-steroidal anti-inflammatory drug		Ibuprofen (Advil)	Diplopia

In addition, Table 2 summarizes the most common or most serious ocular side effects.

**Table 2:** Most common and most serious ocular side effects UTIs: urinary tract infections, SSRIs: selective serotonin reuptake inhibitors, TB: tuberculosis, HIV: human immunodeficiency virus, PPS: pentosan polysulfate sodium, BPH: benign prostatic hyperplasia, IOP: intraocular pressure

Drug class	Drug generic (brand name) or drug class	Used for	Ocular side effects	
			Most common	Most serious
Antiarrhythmic drugs	Amiodarone (Pacerone)	Cardiac arrhythmia	Corneal deposits (69%-100%)	-
Antibiotics	Aminoglycosides	Bacterial infection	Retinal hemorrhages	-
Antibiotics	Fluoroquinolones	UTIs	-	Retinal detachment
Antibiotics	Nalidixic acid (Nevigramon)	UTIs	Reversible sixth nerve palsy	-
Antibiotics	Synthetic penicillin	Ear infection, UTIs	Mild eye redness, dry eyes	-
Antibiotics	Sulfonamides	Bacterial infection	Corneal and conjunctival scarring	-
Antibiotics	Tetracycline (Sumycin) or minocycline (Minocin)	Acne	-	Scleral pigmentation (irreversible)
Antibody-drug conjugates: anti-tumor necrosis factor-alpha inhibitors	Infliximab	Autoimmune diseases such as rheumatoid arthritis and psoriasis	Optic neuritis	Orbital cellulitis, endogenous endophthalmitis
Anticancer drugs: immune checkpoint inhibitors	Pembrolizumab and nivolumab	Lymphoma and melanoma	Dry eye syndrome	Uveitis and scleritis
Antidepressants	Amitriptyline (Elavil) and SSRIs	Depression/anxiety	Dry eye syndrome	-
Anti-estrogen	Tamoxifen (Nolvadex)	Breast cancer	-	Crystalline maculopathy
Antihistamines	Antihistamines	Allergy	Dry eye syndrome	-
Antipsychotics	Phenothiazines: thioridazine (Mellaril) and chlorpromazine (Thorazine)	Schizophrenia/bipolar disorder, hallucinations, and delusions	-	Pigmentary retinopathy
Antitubercular drugs	Rifabutin (Mycobutin)	TB	Turns tears orange-red	-
Antiviral	Cidofovir	HIV	Anterior uveitis	-
Dermatologic agents	Isotretinoin (Accutane)	Acne	Dry eye syndrome	-
Hormones	Levothyroxine (Synthroid)	Hypothyroidism	-	Pseudotumor cerebri in children
Non-steroidal anti-inflammatory drug	Ibuprofen (Advil)	Fever and pain	Dry eye syndrome	-
Other	Elmiron (PPS)	Interstitial cystitis	-	Pigmentary maculopathy
Selective alpha 1 blockers	Tamsulosin (Flomax)	BPH	-	Intraoperative floppy iris syndrome
Steroids	Prednisolone (Pediapred)	Inflammation	High IOP, cataract	-

We conducted a PubMed search to find pertinent case reports, case series, clinical trials, and reviews that detailed the ocular side effects of these drugs. We searched the PubMed database. The keywords used were "ocular toxicity" AND "anticancer medications", "antiarrhythmic drugs", "antibiotics", "antidepressants", "antiepileptic drugs", "antihistamines", "antipsychotics", "antitubercular", "dermatologic agents", "hormones", "non-steroidal anti-inflammatory", "antihypertensive medications", "immunotherapy drugs", "biologic medicines", and "antiviral". Ocular toxicity was added to each medication category independently. The search ended in February 2024. An overview of the ocular adverse effects associated with the most common medications is given in this review article.

## Review

Antiarrhythmic drugs

Amiodarone

Amiodarone is used to treat cardiac arrhythmias and has been classified as a class III antiarrhythmic drug. Its lengthy half-life of roughly 40-55 days and lipid solubility enable it to reach several bodily regions. It is the most widely used antiarrhythmic medication for suppressing ventricular arrhythmias [1]. The amiodarone mechanism of action is proposed to reduce atrioventricular (AV) and sinus node (SN) conduction.

One of the ocular adverse effects of amiodarone is dose- and time-dependent corneal deposits. The most frequent ocular adverse effect, occurring in three stages and affecting 69%-100% of individuals, is corneal deposits. A horizontal line first appears in the cornea in stage 1. A verticillate pattern appears in stages 2 and 3.

According to 25 case reports, there have been ocular side effects [2], with cornea verticillata/vortex keratopathy being the most often described ocular side effect.

Antiepileptic drugs

Topiramate (Topamax)

It is useful not only as a prophylactic measure against migraines but also as an adjuvant or monotherapy for seizure treatment. There have been reports of ocular complications induced by topiramate and elevated intraocular pressure (IOP). It has been suggested that prostaglandin (PG)-mediated ciliary body (CB) swelling, which causes the crystalline lens to migrate forward, is the cause of acute angle closure (ACG) [3]. It commonly happens within the first two weeks of initiating the medication or titrating the dosage.

A number of ocular side effects, including myopic shift, acute angle closure, myokymia, and choroidal effusion syndrome, have been documented. There is a 3% chance of adverse effects [4].

Vigabatrin (Sabril)

Vigabatrin has been used to treat refractory complex partial seizures and infantile spasms. It is not the drug of choice for complex partial seizures due to warnings about permanent visual loss [5]. There is a black box warning that highlights the possibility of progressive and permanent bilateral visual field constriction as well as the possible decline of visual acuity [5] due to the medication's high risk of causing vision loss in 34%-44% of patients who take it [6,7]. The period, which has been proposed to be longer than six months, is connected to the risk of vision loss [8]. However, there is no known risk-free duration or dosage for vigabatrin.

Goldmann kinetic perimetry or automated static perimetry, along with a baseline ophthalmology examination, are required for patients. Furthermore, following two to three months of treatment, patients' responses to standard doses, typically 3 g/day for adults, can be assessed. Finally, patients will need routine ophthalmology reevaluation and repeat perimetry at intervals of three to six months [8].

A decrease in color vision, complex visual perception, and visual acuity was also reported by some patients [8].

Vigabatrin retinal toxicity was initially documented in a case report by Barrett et al. (2014). This report shows electroretinographic (ERG) changes that take place prior to imaging and visual field deterioration. Prior to the retinal nerve fiber layer (RNFL) becoming thinner, on optical coherence tomography (OCT), there was a reduction in maximal ERG [9].

Antimalarial drugs

Hydroxychloroquine (HCQ) and Chloroquine (CQ)

In the USA, it has been used to treat systemic lupus erythematosus (SLE) and rheumatoid arthritis (RA), as well as malaria. Their anti-inflammatory, antithrombotic, and immunomodulatory effects continue to be evident.

CQ stops the proliferation of *Plasmodium* by inhibiting the DNA and RNA polymerase processes, as well as the parasitic ability to utilize hemoglobin. Similarly, by raising pH and hindering hemoglobin breakdown, HCQ disrupts the function of parasitic digesting vacuoles. Furthermore, by blocking neutrophil and eosinophil functions and compromising complement-dependent responses, HCQ can be used as an antirheumatic drug.

For SLE and RA, the typical doses are 200-400 mg/day.

On ocular examination, bilateral bull's eye maculopathy is indicative of CQ and HCQ toxicity. Development of a bull's eye lesion, persistent paracentral or central visual field scotomas, has been seen while using the drug for more than nine months of treatment. Regular monitoring is recommended to avoid permanent vision impairment, as evidenced by reducing sensitivity in the parafoveal point on visual field testing [10].

They change normal physiological function by binding it to melanin and becoming concentrated in the CB, retinal pigment epithelium (RPE), and iris. This results in RPE degenerative changes.

Other ocular side effects include corneal deposits, posterior subcapsular lens opacity, ciliary body dysfunction, and optic disc pallor. 

Accelerated HCQ toxic retinopathy was reported within one year of starting the treatment [10]. While HCQ-induced vortex keratopathy is thought to be dose- and time-dependent, corneal abnormalities are expected to increase in frequency if high-dose chemotherapy is used as an adjuvant [11].

Hydroxychloroquine screening recommendations: The American Academy of Ophthalmology (AAO) published screening recommendations for hydroxychloroquine patients in 2011. In addition to visual field analysis, fundus autofluorescence, and ERG (multifocal electroretinogram), the guidelines suggested the use of OCT macula (ocular coherence tomography). According to the guidelines, patients should be examined both when they first start using the medication and five years later. Patients with a cumulative overdosage of more than 1000 g, those with renal or hepatic dysfunction, those with concomitant maculopathy, and those on dosages greater than 6.5 mg/kg/day of ideal body weight or more than 400 mg/day were recommended to undergo screening more frequently [12]. In 2016, the updated screening guidelines considered age and hepatic impairment as minor risk factors and emphasized real body weight as a stronger predictor of developing toxicity, with a recommended dosage below 5 mg/kg/day of actual body weight. After the five-year baseline assessment, if the patient has no significant risk factors, they should undergo an annual screening examination. In addition to the screening evaluation, OCT, and visual fields, ERG and fundus autofluorescence are still recommended when needed [13].

Antitubercular drugs

Ethambutol

Ethambutol is a medication that is used to treat tuberculosis (TB), and it is typically administered in combination with at least one other medication. Ethambutol is a medication that is used as an antibiotic that inhibits metabolite synthesis and impairs cell metabolism in susceptible mycobacteria. It prevents bacterial growth, which refers to bacteriostatic. It is frequently taken in conjunction with pyrazinamide, rifampin, and isoniazid as part of a four-drug regimen.

Ethambutol doses range from 15 to 25 mg/kg for daily dosing strategies to 50 mg/kg twice weekly, depending on the regimen. The standard course of treatment for TB requires an ethambutol dose range of eight weeks. Ethambutol use is linked to several ocular side effects, the most severe of which is optic neuritis, primarily retrobulbar neuritis [14]. Ethambutol has been proven to be an optic nerve toxin that causes dose-dependent, bilateral, and progressive damage [15] in addition to irreversible changes. In animals receiving 35, 25, and 15 mg/kg/day of ethambutol, the incidence of damaged nerves at two months of therapy is 18%, 6%, and 1%, respectively [16]. There have also been reports of changes in color vision and visual field defects in ocular adverse events.

A case of miliary TB (pulmonary and cerebral) was reported in which a 16-year-old female patient complained of bilateral vision reduction for 10 -12 days. After five months of first-line antitubercular treatment with rifampicin (450 mg/day), ethambutol (800 mg/day), pyrazinamide (1500 mg/day), and isonicotinylhydrazide (INH) (300 mg/day), she noticed reduced vision, and she underwent a visual field examination (central 30-2, SITA-Fast). The results showed incomplete left homonymous hemianopia along with additional defects in the right eye inferior quadrants. Ethambutol was stopped when the clinical presentation suggested ethambutol toxicity, and she was recommended to follow up. Her visual acuity had recovered to normal at the three-month follow-up, and she was referred to her primary care physician [17].

Phenytoin

Phenytoin is a medication that is used to treat tuberculosis and as an anticonvulsant. Nystagmus, diplopia, and acute bilateral visual loss have been reported as ocular side effects of using phenytoin.

A 20-year-old male patient with focal epilepsy presented with acute bilateral visual loss while taking phenytoin. After stopping using phenytoin for 84 hours, his visual symptoms completely resolved. This case demonstrates that reversible visual loss can result from acute phenytoin toxicity [18].

Rifamycin

Rifabutin, which is an antibiotic used to prevent *Mycobacterium avium* complex (MAC), is used to treat TB in human immunodeficiency virus (HIV)-positive patients [19]. Rifabutin was commonly used in the HIV pandemic during the second and third decades [20].

Rifamycin includes rifampin, rifapentine, and rifabutin.

Rifampin is intensely red, which turns tears orange-red. In addition, tears stain soft-contact lenses permanently. Other ocular side effects include hypopyon, anterior uveitis, intermediate uveitis, panuveitis, and retinal vasculitis [21].

Antibiotics

Aminoglycosides

Aminoglycosides are bactericidal antibiotics that are widely used against gram-negative bacteria due to their reduced cost. The most common serious side effects are ototoxicity [22] and nephrotoxicity [23]. Retinal hemorrhages, retinal edema, cotton-wool spots, arteriolar narrowing, venous beading, rubeosis iridis, neovascular granuloma, pigmentary retinopathy, and optic atrophy can all be caused by high doses of aminoglycosides. Methylparaben, propylparaben, and edetate disodium are three preservatives that may contribute to toxicity in combination. It has been reported that the most toxic aminoglycoside antibiotic is gentamicin [24].

Fluoroquinolones

Fluoroquinolones are broad-spectrum antibiotics that have been used to treat a variety of infections, such as urinary tract infections and pneumonia. Its major topical use is bacterial keratitis. A recent study found that patients using oral fluoroquinolones had a 4.5-fold increased risk of retinal detachment, which manifests within five days of starting the treatment [25,26]. However, other studies showed no retinal detachment increased risk [27,28].

Nalidixic Acid

Nalidixic acid, an oral quinolone antibacterial agent that is used for urinary tract infections, works by inhibiting bacteria. First-generation quinolones such as nalidixic acid are used to treat urinary tract infections, targeting gram-negative bacteria, and have been shown to have significant drug interactions with warfarin (Coumadin).

Nalidixic acid has been reported to cause headaches (believed to be caused by increased fluid pressure), vision disturbances, color vision defects, and swollen optic nerves, leading to papilledema and reversible sixth nerve palsy [29].

Synthetic Penicillin (Aminopenicillins)

Aminopenicillins are the most commonly used penicillins to treat infections such as ear infections, urinary tract infections, and pneumonia. Amoxicillin and ampicillin are synthetic penicillins that can cause mild eye redness, itching, and dry eyes. They are frequently linked to toxic epidermal necrolysis and Steven-Johnson syndrome (SJS) [30].

Sulfonamides

Sulfonamides are used to treat bacterial infections. Sulfonamides, when used topically and systemically, result in corneal and conjunctival scarring in Steven-Johnson syndrome (SJS) [31]. Due to its frequent overlap with other rheumatic diseases, which can lead to misdiagnosis or neglect, up to four million Americans may be affected by SJS. SJS is an autoimmune disease mainly affecting women. This condition is caused by inflammation in the lacrimal and salivary glands. Unfortunately, many patients are diagnosed late, which greatly increases treatment challenges.

Tetracycline and Minocycline

Tetracycline is used to treat respiratory infections, including pneumonia. Tetracycline is used against gram-negative bacteria. Tetracycline and minocycline have been used to treat severe acne. It has been documented that minocycline therapy can cause pigmentation of the skin, nails, bones, mouth, and eyes [32,33]. While pigmentation of the skin and mucous membranes can be reversed, eye pigmentation is usually irreversible [33]. In addition, extended use has been linked to idiopathic intracranial hypertension, which can occasionally result in permanent vision loss [34].

Antiviral drugs

Cidofovir

The most prevalent ocular side effect is anterior uveitis [35].

Ganciclovir

Ganciclovir has been reported with retinal detachment, eye hemorrhage, macular edema, vitreous floaters, eye pain, visual impairment, vitreous disorders, conjunctivitis, cataracts, and dry eyes [36-39].

Anticancer drugs

Immune Checkpoint Inhibitors

Immunotherapy has recently been used to treat a number of malignancies, such as lymphoma and melanoma. The immunotherapy mechanism of action is by boosting T lymphocytes' immune response to tumor cells.

Examples of anti-programmed death ligand 1 (anti-PD1) are pembrolizumab and nivolumab, which indicate that T-cells can kill tumor cells by blocking PD-1 [40-43].

The most common ocular side effect was dry eye syndrome in 15% of patients [44]. Additional ocular adverse effects include blepharitis and conjunctivitis. Additionally, myasthenia gravis has been reported in 4% of patients [44]. Other side effects reported were uveitis and scleritis in 13% of patients [44].

Tyrosine Kinase Inhibitors

Tyrosine kinase inhibitors inhibit the kinase signaling pathways that limit tumor cell proliferation, differentiation, and survival, ultimately resulting in tumor cell death [45].

Examples of tyrosine kinase inhibitors are imatinib and ripretinib, which are derivatives of the platelet growth factor receptor [45]. Imatinib has been shown to cause epiphora, periorbital edema, and subconjunctival hemorrhages [46,47].

Vascular Endothelial Growth Factor (VEGF) Inhibitors

The VEGF inhibitor (endothelial cell growth factor) mechanism of action works by blocking the VEGF pathway, which prevents tumor growth and has an anti-apoptotic effect on endothelial cells [48]. Bevacizumab was the first VEGF inhibitor to be approved. Ocular hemorrhage has been documented as a side effect of bevacizumab [49].

Cytotoxic Agents

Cisplatin is a cytotoxic agent that results in cell death by enhancing reactive oxygen species by causing DNA damage [50]. The main ocular manifestations include blepharitis, conjunctivitis, visual field defects, eyelid swelling, ocular pain, retinal ischemia, and retinal toxicity [51-53].

Immuno-Based Cancer Drugs

Immunotherapy such as rituximab has been employed since 1997. Rituximab is an IgG1 monoclonal antibody (mAb) against CD20, which is predominately found in B-cells. It induces apoptosis of the tumor cells. Its main focus was developing progressive multifocal leukoencephalopathy (PML), which is an infectious demyelinating central nervous system disease caused by the polyomavirus John Cunningham (JC) virus linked to a high rate of morbidity and mortality [54]. Patients who develop PML develop visual complaints and visual field defects due to the involvement of the posterior visual pathways within the parietal-occipital lobes.

Rituximab ocular toxicities reported are retinal necrosis [55], macular edema [56], transient IOP elevations [57], iridocyclitis, and cytomegalovirus (CMV) retinitis [58]. Other ocular toxicities include conjunctivitis [59], visual disturbance [57], and periorbital edema [54,60-62].

Antibody-drug conjugates

Anti-tumor Necrosis Factor (TNF)-Alpha Inhibitors

A crucial first step in triggering the immune response is TNF-alpha. TNF-alpha inhibitors such as infliximab block the TNF-alpha and prevent immune response activation. They are used to treat psoriasis, rheumatoid arthritis, and psoriatic arthritis. They also reduce the flares of many autoimmune diseases, such as sarcoidosis, Behcet's disease, and inflammatory bowel disease [63].

Infliximab ocular side effects include retinal vein thrombosis [64], bilateral rosacea-like keratopathy [65], optic neuritis [66], CMV retinitis [67], toxoplasmic chorioretinitis, orbital cellulitis, and endogenous endophthalmitis [65,67-69].

Interleukin-6 (IL-6) Receptor Inhibitors

IL-6 inhibitors have been used to treat rheumatoid arthritis, giant cell arthritis, and juvenile idiopathic arthritis. Tocilizumab, an example of IL-6, has been demonstrated to induce viral conjunctivitis [70], ophthalmic herpes zoster infection [71], human T-lymphotropic virus type 1 (HTLV-1) uveitis [70,72], and fulminant bilateral papilledema [73].

Selective alpha 1 blockers

Tamsulosin (Flomax)

Tamsulosin is a selective alpha 1A antagonist that is frequently prescribed to treat benign prostatic hyperplasia (BPH).

While alpha-blockers are mainly used by men for BHP, women may also be at risk of intraoperative floppy iris syndrome (IFIS) due to their use to treat bladder problems and hypertension. The mechanism of action is thought to be blockage of the alpha 1A receptor, which causes iris dilator relaxation, making the procedure's agents less effective. IFIS is primarily observed in cataract surgery, and it is also occasionally observed in glaucoma surgery [74,75].

The IFIS associated with these drugs may be relieved by stopping them prior to surgery [75], but this may not always be the case. IFIS has been reported in individuals who stopped treatment up to three years or even nine months prior to cataract surgery; at least, this has been documented with tamsulosin. Mydriatic drugs are used to dilate the pupil, which is a precautionary measure to lower the risk of complications during cataract surgery (two days prior to surgery, 1% of topical atropine sulfate should be applied three times a day) [75].

Antihypertensive drugs

Angiotensin-Converting Enzyme (ACE) Inhibitors

ACE inhibitors, such as lisinopril, have been used to treat hypertension. The most common side effects reported are decreased vision, photophobia, conjunctivitis, and retinal hemorrhage [76].

Beta-Blockers

Metoprolol is an example of a beta-blocker. Ocular side effects include dry eye, conjunctivitis, corneal epithelial damage, and lower IOP [77]. 

Calcium Channel Blockers

Amlodipine: Amlodipine has been linked to glaucoma progression and may be a factor that leads to open-angle glaucoma [78,79] or normal tension glaucoma [80,81].

Anti-diabetic drugs

Sodium-Glucose Cotransporter 2 (SGLT2) Inhibitors (Canagliflozin)

There have been cases of vitreous hemorrhage [82] associated with canagliflozin.

GLP-1 Agonists (Semaglutide)

Semaglutide has been linked with blurry vision, worsening diabetic retinopathy, and macular complications [83-85]. In addition, it has been associated with an increased risk of non-arteritic anterior ischemic optic neuropathy [86].

Antidepressants

In the USA, 5.3% of adult men and 8.7% of adult women suffer from depression [87]. A total of 16.2 million US adults, or 6.7% of the population, suffered at least one major depressive episode in 2016, according to the National Institute of Mental Health. An insightful study conducted in 2020 by Ettman et al. revealed that during the COVID-19 pandemic, the prevalence of depression symptoms in the USA was more than three times higher than it was prior to the pandemic [88]. In addition, the COVID-19 pandemic increased mental health illness by 6% globally [89]. Tricyclic antidepressants (TCAs) such as amitriptyline and selective serotonin reuptake inhibitors (SSRIs) cause a variety of anticholinergic adverse effects, including dilated pupils, which causes light sensitivity, dry eyes due to reduced tear production [90,91], cataract [92,93], and difficulty with lens accommodation [94], which makes it hard to focus at near work and worsens acute closed-angle glaucoma [95]. TCAs can cause glaucomatous attacks in those who are susceptible [95]. This is mostly because TCAs have cholinergic action, which might lead to mydriases and cycloplegia.

In addition, SSRIs have been linked to angle-closure glaucoma [96], primary open-angle glaucoma [97], cataract [93], dry eye [90], mydriasis, and elevated IOP. When patients are using antidepressants, a gonioscopy examination should be performed to rule out any angle closure since it aids in early identification.

Non-steroidal anti-inflammatory drug

Ibuprofen

Ibuprofen has been used for fever and pain. Prolonged use of ibuprofen causes disturbance of color vision, xerosis, diplopia [98], blurring of vision, optic neuritis [99], and permanent visual loss [99].

Psychiatric medications

Atypical Antipsychotics

Olanzapine: It has been documented that olanzapine causes acute angle closure [100] and oculogyric crisis [101]. Furthermore, it has been reported to be associated with retinal vein occlusion [102,103] and periorbital edema [104].

Mood Stabilizers

Lithium: Lithium has been reported to cause skew deviation and downbeat nystagmus [105]. Furthermore, it contributed to a decrease in tear film break-up time due to dry eye [106].

Phenothiazines

Psychotropic medications such as thioridazine (Mellaril) and chlorpromazine (Thorazine), which are used to treat depression, anxiety, and other behavioral disorders, are risky at 800 mg/dL because of their dose-dependent toxicity [107]. By attaching to melanin granules, Thorazine and Mellaril are accumulated in the uveal tissue and RPE. Chlorpromazine toxicity has been reported to be dose-dependent [108]. Abnormal eyelid pigmentation; interpalpebral conjunctiva, cornea, and anterior lens capsule; and anterior and posterior subcapsular cataracts may develop and are typical side effects of high-dose chlorpromazine (Thorazine) therapy [109]. Nonetheless, chlorpromazine-induced pigmentary retinopathy is uncommon, if it occurs at all. However, acute thioridazine retinopathy has been reported [107]. Nyctalopia, or night blindness, which is a sign of an underlying disease such as a retinal or lens problem, is an example of an ocular adverse effect [110]. Any disorder that interferes with the rod's ability to detect light is known as night blindness. In other words, night blindness is having less vision at night and in dimly illuminated environments [110]. The rods in the retina merely detect light, which enables you to see in grayscale. For instance, a cataract may block the light, making it difficult for rods to detect light correctly.

Other ocular side effects include brown vision, decreased vision, and salt and pepper fundus, leading to widespread loss of RPE and choriocapillaris [110]. Brown vision results in a brown hue [109].

Notably, thioridazine was withdrawn worldwide in 2005 due to its cardiac arrhythmia effects that cause excessive death rates [111].

Antihistamines

Antihistamines have ocular side effects and properties that are anticholinergic. Patients with narrow-angle glaucoma who take antihistamines experience blurry vision, redness, halos around light objects, and pain. Mydriasis (pupil dilation), dry eye [112,113], keratitis sicca, intolerance to contact lenses, decreased accommodation, and other ocular side effects are also possible.

Antihistamines can cause mydriasis, anisocoria, decreased accommodation, and blurred vision due to their weak atropine-like action [114]. The following symptoms can be summed up: hyperthermia, confusion, dry skin or membranes, mydriasis, flushing due to vasodilation, urinary tract retention, and tachycardia [114].

Dermatologic agents

Accutane (Isotretinoin)

Meibomian gland dysfunction, decreased dark adaptation, blepharoconjunctivitis, corneal opacities, keratitis, photophobia, teratogenic ocular abnormalities, and night vision disturbances have been reported as ocular side effects of Accutane. Dry eye is the most commonly reported ocular side effect [115]. Having trouble wearing contact lenses might be caused by dryness and irritation. Visual disturbances are other symptoms that could occur due to pseudotumor cerebri. In addition, ocular pseudo-myasthenic reaction has been reported [116]. Dermatologists should constantly inquire about any vision symptoms; their patients may have both isotretinoin therapy and should be referred to an ophthalmologist if necessary.

According to a systemic review, changes in vision, dry eyes, and ocular inflammatory conditions are among the reported ocular adverse effects [117].

Minocycline and Tetracycline

For minocycline and tetracycline, please refer to the section on "Antibiotics."

Steroids

Steroids are frequently used for a variety of dermatologic conditions, including skin disorders and allergies, inflammatory conditions, autoimmune conditions, and vesiculobullous disorders. In addition, steroids have also been used to treat pulmonary diseases such as asthma as inhalers. It is noteworthy that medications administered through any route, including topical, oral, intramuscular, or intravenous, may affect the eye. Cataracts and high IOP are the two major complications of steroid use [118]. Predominantly, posterior subcapsular cataracts occur [119]. Later, it may affect the anterior subcapsular lens. It progresses quickly and shows symptoms in a matter of weeks or months. Patients treated for less than four years with <10 mg/day of prednisolone often do not acquire cataracts [120]. Steroid use for at least two weeks is typically associated with high IOP, which refers to steroid responders. They typically have no symptoms and can be reversed upon discontinuation. Topical ocular or periocular steroids are more frequently associated with high IOP than systemic steroids.

Anti-estrogen

Tamoxifen can be used in women and men to treat metastatic breast cancer. Crystalline maculopathy may result from Tamoxifen [121].

Hormones

Levothyroxine (L-T4) is used to treat goiters, thyroid cancer, and hypothyroidism. It is also used five days following thyroidectomy; for benign thyroid pathology, 87% of patients started taking L-T4.

The synthetic hormone levothyroxine, also known as Synthroid, is used as a replacement if the thyroid is eliminated entirely. Levothyroxine treatment has been reported to be linked to an increased risk of cardiac arrhythmia [122]. Long-term usage of levothyroxine has been associated with eyelid hyperemia, visual hallucinations, idiopathic intracranial hypertension [123], and pseudotumor cerebri in children [124].

Other

Elmiron

Elmiron, also known as pentosan polysulfate sodium (PPS), is the only oral medication authorized to treat interstitial cystitis (IC)-related bladder pain and discomfort [125]. It has been reported that long-term PPS use is linked to a higher prevalence of vision-threatening pigmented maculopathy [126]. The mechanism of action is thought to involve toxicity to the RPE layer or potential interference with the glycosaminoglycan-based interphotoreceptor matrix [127].

According to a retrospective study, the daily dose of Elmiron ranges from 150 to 592 mg, and the period of pharmaceutical use ranges from three to 22 years. The most typical visual symptoms include metamorphopsia, blurred vision, and prolonged dark adaptation. Furthermore, fundus examination demonstrated interspersed pale-yellow deposits and hyperpigmented macular spots [127]. Therefore, it is recommended that every patient who has been taking PPS get ocular screening [128].

Bisphosphonates, Linezolid, and Isoniazid

It has been reported that the oxazolidinone antibiotic linezolid causes optic nerve damage. It is the first member of a novel synthetic class of antimicrobials that exhibits activity against multidrug-resistant tubercle bacillus, *Streptococcus*, and methicillin-resistant *Staphylococcus* [129]. 

Isoniazid and ethambutol: Isoniazid and ethambutol have been used as antituberculosis drugs. Especially when used as an adjuvant medication to ethambutol, ocular toxicities such as optic neuropathy and optic atrophy have been documented [130-132].

Bisphosphonates: Oral bisphosphonates have been used to treat osteoporosis. Examples of oral bisphosphonates are alendronate, risedronate, and ibandronate. They have been associated with conjunctivitis, eyelid edema, optic or retrobulbar neuritis, periorbital edema, cranial nerve palsy, and ptosis [133]. Other ocular side effects reported are uveitis and scleritis [133,134].

## Conclusions

Ocular side effects are very straightforward; by taking a good history of the medication lists and recognizing their side effects, eye care physicians could rule out a lot of signs and symptoms. Some ocular side effects require early recognition to prevent irreversible vision loss, so we should be very cautious of these medications. These medications require home monitoring (e.g., for hydroxychloroquine) and regular monitoring of side effects by an ophthalmologist if the patient is at high risk of getting the ocular side effects. Other medications, such as tamoxifen, could lead to permeant crystalline maculopathy; others, such as vigabatrin, require immediate attention due to its progressive and permanent bilateral visual field constriction.

## References

[1] King GS, Goyal A, Grigorova Y, Patel P, Hashmi MF (2024). Antiarrhythmic medications. StatPearls Publishing, Treasure Island.

[2] Alshehri M, Joury A (2020). Ocular adverse effects of amiodarone: a systematic review of case reports. Optom Vis Sci.

[3] Rhee DJ, Goldberg MJ, Parrish RK (2001). Bilateral angle-closure glaucoma and ciliary body swelling from topiramate. Arch Ophthalmol.

[4] Lobo S, Lobo GJ (2024). Topiramate-induced ocular complications: case series. Rom J Ophthalmol.

[5] Bruni J, Guberman A, Vachon L, Desforges C (2000). Vigabatrin as add-on therapy for adult complex partial seizures: a double-blind, placebo-controlled multicentre study. The Canadian Vigabatrin Study Group. Seizure.

[6] Riikonen R, Rener-Primec Z, Carmant L (2015). Does vigabatrin treatment for infantile spasms cause visual field defects? An international multicentre study. Dev Med Child Neurol.

[7] Maguire MJ, Hemming K, Wild JM, Hutton JL, Marson AG (2010). Prevalence of visual field loss following exposure to vigabatrin therapy: a systematic review. Epilepsia.

[8] Krauss GL (2009). Evaluating risks for vigabatrin treatment. Epilepsy Curr.

[9] Barrett D, Yang J, Sujirakul T, Tsang SH (2014). Vigabatrin retinal toxicity first detected with electroretinographic changes: a case report. J Clin Exp Ophthalmol.

[10] Mohapatra A, Gupta P, Ratra D (2024). Accelerated hydroxychloroquine toxic retinopathy. Doc Ophthalmol.

[11] Savage DE, Plotnik R, Wozniak RA (2020). Short-term, high-dose hydroxychloroquine corneal toxicity. Am J Ophthalmol Case Rep.

[12] Marmor MF, Kellner U, Lai TY, Lyons JS, Mieler WF (2011). Revised recommendations on screening for chloroquine and hydroxychloroquine retinopathy. Ophthalmology.

[13] Marmor MF, Kellner U, Lai TY, Melles RB, Mieler WF (2016). Recommendations on screening for chloroquine and hydroxychloroquine retinopathy (2016 revision). Ophthalmology.

[14] Koul PA (2015). Ocular toxicity with ethambutol therapy: timely recaution. Lung India.

[15] Ezer N, Benedetti A, Darvish-Zargar M, Menzies D (2013). Incidence of ethambutol-related visual impairment during treatment of active tuberculosis. Int J Tuberc Lung Dis.

[16] Huang SP, Chien JY, Tsai RK (2015). Ethambutol induces impaired autophagic flux and apoptosis in the rat retina. Dis Model Mech.

[17] Mehta S (2019). Patterns of ethambutol ocular toxicity in extended use therapy. Cureus.

[18] Chew SH, Looi I, Lo YL, Lim KS (2018). Phenytoin toxicity presenting with acute visual loss and acute delirium, a case report. Neurol Asia.

[19] Phillips MC, Wald-Dickler N, Loomis K, Luna BM, Spellberg B (2020). Pharmacology, dosing, and side effects of rifabutin as a possible therapy for antibiotic-resistant Acinetobacter infections. Open Forum Infect Dis.

[20] Hennig S, Svensson EM, Niebecker R (2016). Population pharmacokinetic drug-drug interaction pooled analysis of existing data for rifabutin and HIV PIs. J Antimicrob Chemother.

[21] Smith WM, Reddy MG, Hutcheson KA, Bishop RJ, Sen HN (2012). Rifabutin-associated hypopyon uveitis and retinal vasculitis with a history of acute myeloid leukemia. J Ophthalmic Inflamm Infect.

[22] Guthrie OW (2008). Aminoglycoside induced ototoxicity. Toxicology.

[23] Wargo KA, Edwards JD (2014). Aminoglycoside-induced nephrotoxicity. J Pharm Pract.

[24] Thomas T, Galiani D, Brod RD (2001). Gentamicin and other antibiotic toxicitiy. Ophthalmol Clin North Am.

[25] Etminan M, Forooghian F, Brophy JM, Bird ST, Maberley D (2012). Oral fluoroquinolones and the risk of retinal detachment. JAMA.

[26] Fife D, Zhu V, Voss E, Levy-Clarke G, Ryan P (2014). Exposure to oral fluoroquinolones and the risk of retinal detachment: retrospective analyses of two large healthcare databases. Drug Saf.

[27] Chui CS, Wong IC, Wong LY, Chan EW (2015). Association between oral fluoroquinolone use and the development of retinal detachment: a systematic review and meta-analysis of observational studies. J Antimicrob Chemother.

[28] Alves C, Penedones A, Mendes D, Batel Marques F (2016). A systematic review and meta-analysis of the association between systemic fluoroquinolones and retinal detachment. Acta Ophthalmol.

[29] Fraunfelder FT, Fraunfelder FW (2020). Drug-induced ocular side effects: clinical ocular toxicology.

[30] Sethuraman G, Sharma VK, Pahwa P, Khetan P (2012). Causative drugs and clinical outcome in Stevens Johnson syndrome (SJS), toxic epidermal necrolysis (TEN), and SJS-TEN overlap in children. Indian J Dermatol.

[31] Okonkwo SN, Ezemba U (2022). Ocular complications of Stevens - Johnson syndrome and toxic epidermal necrolysis at a tertiary hospital in southern Nigeria. J Med Dent Sci Res.

[32] Santaella RM, Fraunfelder FW (2007). Ocular adverse effects associated with systemic medications : recognition and management. Drugs.

[33] Haskes C, Shea M, Imondi D (2017). Minocycline-induced scleral and dermal hyperpigmentation. Optom Vis Sci.

[34] Wall M, Falardeau J, Fletcher WA (2015). Risk factors for poor visual outcome in patients with idiopathic intracranial hypertension. Neurology.

[35] Scott RA, Pavesio C (2000). Ocular side-effects from systemic HPMPC (Cidofovir) for a non-ocular cytomegalovirus infection. Am J Ophthalmol.

[36] Faulds D, Heel RC (1990). Ganciclovir. A review of its antiviral activity, pharmacokinetic properties and therapeutic efficacy in cytomegalovirus infections. Drugs.

[37] Abdallah EA, Al-Helal B, Asad R (2014). Ganciclovir-induced acute liver injury in a patient with lupus nephritis. Saudi J Kidney Dis Transpl.

[38] Izzedine H, Launay-Vacher V, Deray G (2005). Antiviral drug-induced nephrotoxicity. Am J Kidney Dis.

[39] Ernst ME, Franey RJ (1998). Acyclovir- and ganciclovir-induced neurotoxicity. Ann Pharmacother.

[40] Kwok G, Yau TC, Chiu JW, Tse E, Kwong YL (2016). Pembrolizumab (Keytruda). Hum Vaccin Immunother.

[41] Flynn JP, Gerriets V (2024). Pembrolizumab. https://pubmed.ncbi.nlm.nih.gov/31536223/.

[42] Buchbinder EI, Desai A (2016). CTLA-4 and PD-1 pathways: similarities, differences, and implications of their inhibition. Am J Clin Oncol.

[43] Finkelmeier F, Waidmann O, Trojan J (2018). Nivolumab for the treatment of hepatocellular carcinoma. Expert Rev Anticancer Ther.

[44] Mazharuddin AA, Whyte AT, Gombos DS, Patel N, Razmandi A, Chaudhry AL, Al-Zubidi NS (2022). Highlights on ocular toxicity of immune checkpoint inhibitors at a US tertiary cancer center. J Immunother Precis Oncol.

[45] Ebrahimi N, Fardi E, Ghaderi H (2023). Receptor tyrosine kinase inhibitors in cancer. Cell Mol Life Sci.

[46] Fraunfelder FW, Solomon J, Druker BJ, Esmaeli B, Kuyl J (2003). Ocular side-effects associated with imatinib mesylate (Gleevec). J Ocul Pharmacol Ther.

[47] Kheir WJ, Sniegowski MC, El-Sawy T, Li A, Esmaeli B (2014). Ophthalmic complications of targeted cancer therapy and recently recognized ophthalmic complications of traditional chemotherapy. Surv Ophthalmol.

[48] Cardones AR, Banez LL (2006). VEGF inhibitors in cancer therapy. Curr Pharm Des.

[49] Taugourdeau-Raymond S, Rouby F, Default A, Jean-Pastor MJ (2012). Bevacizumab-induced serious side-effects: a review of the French pharmacovigilance database. Eur J Clin Pharmacol.

[50] Brozovic A, Ambriović-Ristov A, Osmak M (2010). The relationship between cisplatin-induced reactive oxygen species, glutathione, and BCL-2 and resistance to cisplatin. Crit Rev Toxicol.

[51] Kwan AS, Sahu A, Palexes G (2006). Retinal ischemia with neovascularization in cisplatin related retinal toxicity. Am J Ophthalmol.

[52] Wilding G, Caruso R, Lawrence TS, Ostchega Y, Ballintine EJ, Young RC, Ozols RF (1985). Retinal toxicity after high-dose cisplatin therapy. J Clin Oncol.

[53] Wu HM, Lee AG, Lehane DE, Chi TL, Lewis RA (1997). Ocular and orbital complications of intraarterial cisplatin. A case report. J Neuroophthalmol.

[54] White MK, Khalili K (2011). Pathogenesis of progressive multifocal leukoencephalopathy--revisited. J Infect Dis.

[55] Schuler S, Brunner M, Bernauer W (2016). Rituximab and acute retinal necrosis in a patient with scleromalacia and rheumatoid arthritis. Ocul Immunol Inflamm.

[56] Bussone G, Kaswin G, de Menthon M, Delair E, Brézin AP, Guillevin L (2010). Macular oedema following rituximab infusion in two patients with Wegener's granulomatosis. Clin Exp Rheumatol.

[57] Hashida N, Ohguro N, Nishida K (2012). Efficacy and complications of intravitreal rituximab injection for treating primary vitreoretinal lymphoma. Transl Vis Sci Technol.

[58] Dogra M, Bajgai P, Kumar A, Sharma A (2018). Progressive outer retinal necrosis after rituximab and cyclophosphamide therapy. Indian J Ophthalmol.

[59] Marinella MA (2007). Bilateral conjunctivitis due to rituximab. Ann Pharmacother.

[60] Agustoni F, Platania M, Vitali M (2014). Emerging toxicities in the treatment of non-small cell lung cancer: ocular disorders. Cancer Treat Rev.

[61] Renouf DJ, Velazquez-Martin JP, Simpson R, Siu LL, Bedard PL (2012). Ocular toxicity of targeted therapies. J Clin Oncol.

[62] al-Tweigeri T, Nabholtz JM, Mackey JR (1996). Ocular toxicity and cancer chemotherapy. A review. Cancer.

[63] Cordero-Coma M, Sobrin L (2015). Anti-tumor necrosis factor-α therapy in uveitis. Surv Ophthalmol.

[64] Vergou T, Moustou AE, Maniateas A, Stratigos AJ, Katsambas A, Antoniou C (2010). Central retinal vein occlusion following infliximab treatment for plaque-type psoriasis. Int J Dermatol.

[65] Fasci-Spurio F, Thompson A, Madill S, Koay P, Mansfield D, Satsangi J (2014). Sight-threatening keratopathy complicating anti-TNF therapy in Crohn's disease: a case report. Inflamm Bowel Dis.

[66] Lin EJ, Reddy S, Shah VV, Wu JJ (2018). A review of neurologic complications of biologic therapy in plaque psoriasis. Cutis.

[67] Haerter G, Manfras BJ, de Jong-Hesse Y, Wilts H, Mertens T, Kern P, Schmitt M (2004). Cytomegalovirus retinitis in a patient treated with anti-tumor necrosis factor alpha antibody therapy for rheumatoid arthritis. Clin Infect Dis.

[68] Roos JC, Ostor AJ (2006). Orbital cellulitis in a patient receiving infliximab for ankylosing spondylitis. Am J Ophthalmol.

[69] Agarwal PK, Gallaghar M, Murphy E, Virdi M (2007). Endogenous endophthalmitis in a rheumatoid patient on tumor necrosis factor alpha blocker. Indian J Ophthalmol.

[70] Terada Y, Kamoi K, Ohno-Matsui K, Miyata K, Yamano C, Coler-Reilly A, Yamano Y (2017). Treatment of rheumatoid arthritis with biologics may exacerbate HTLV-1-associated conditions: a case report. Medicine (Baltimore).

[71] Roux C, Breuil V, Albert C (2011). Ophthalmic herpes zoster infection in patients with rheumatoid arthritis who were treated with tocilizumab. J Rheumatol.

[72] Calvo-Río V, Santos-Gómez M, Calvo I (2017). Anti-interleukin-6 receptor tocilizumab for severe juvenile idiopathic arthritis-associated uveitis refractory to anti-tumor necrosis factor therapy: a multicenter study of twenty-five patients. Arthritis Rheumatol.

[73] Burstzyn L, Levin S, Rotenberg B, Van Hooren T, Leung A, Berard R, Ardelean DS (2017). Fulminant bilateral papilloedema during low-dose steroid taper in a child with systemic idiopathic arthritis treated with tocilizumab. Clin Exp Rheumatol.

[74] Tobaiqy M, Aalam W, Banji D, Al Haleem EN (2021). Intraoperative floppy iris syndrome induced by tamsulosin: the risk and preventive strategies. Middle East Afr J Ophthalmol.

[75] Bell CM, Hatch WV, Fischer HD (2009). Association between tamsulosin and serious ophthalmic adverse events in older men following cataract surgery. JAMA.

[76] Montoro de Francisco A, García-Luque A, Fernández M, Puerro M (2012). Side effects of angiotensin converting enzyme inhibitors and angiotensin II receptor antagonists: are we facing a new syndrome. Am J Cardiol.

[77] Petounis AD, Akritopoulos P (1989). Influence of topical and systemic beta-blockers on tear production. Int Ophthalmol.

[78] Lehrer S, Rheinstein PH (2024). Amlodipine increases risk of primary open-angle glaucoma. Clin Hypertens.

[79] Vergroesen JE, Schuster AK, Stuart KV (2023). Association of systemic medication use with glaucoma and intraocular pressure: the European Eye Epidemiology Consortium. Ophthalmology.

[80] Kastner A, Stuart KV, Montesano G (2023). Calcium channel blocker use and associated glaucoma and related traits among UK Biobank participants. JAMA Ophthalmol.

[81] Gangwani RA, Lee JW, Mo HY (2015). The correlation of retinal nerve fiber layer thickness with blood pressure in a Chinese hypertensive population. Medicine (Baltimore).

[82] Dorsey-Treviño EG, González-González JG, Alvarez-Villalobos N (2020). Sodium-glucose cotransporter 2 (SGLT-2) inhibitors and microvascular outcomes in patients with type 2 diabetes: systematic review and meta-analysis. J Endocrinol Invest.

[83] Cigrovski Berkovic M, Strollo F (2023). Semaglutide-eye-catching results. World J Diabetes.

[84] Bracha P, Johnson W, Chu S, Davison J (2024). Reversible bilateral central scotoma under scotopic conditions associated with oral semaglutide. Am J Ophthalmol Case Rep.

[85] Sharma A, Parachuri N, Kumar N, Saboo B, Tripathi HN, Kuppermann BD, Bandello F (2022). Semaglutide and the risk of diabetic retinopathy-current perspective. Eye (Lond).

[86] Hathaway JT, Shah MP, Hathaway DB (2024). Risk of nonarteritic anterior ischemic optic neuropathy in patients prescribed semaglutide. JAMA Ophthalmol.

[87] Abe RY, Silva LN, Silva DM, Vasconcellos JP, Costa VP (2021). Prevalence of depressive and anxiety disorders in patients with glaucoma: a cross-sectional study. Arq Bras Oftalmol.

[88] Ettman CK, Abdalla SM, Cohen GH, Sampson L, Vivier PM, Galea S (2020). Prevalence of depression symptoms in US adults before and during the COVID-19 pandemic. JAMA Netw Open.

[89] Wu T, Jia X, Shi H, Niu J, Yin X, Xie J, Wang X (2021). Prevalence of mental health problems during the COVID-19 pandemic: a systematic review and meta-analysis. J Affect Disord.

[90] Acan D, Kurtgoz P (2017). Influence of selective serotonin reuptake inhibitors on ocular surface. Clin Exp Optom.

[91] Zhang X, Yin Y, Yue L, Gong L (2019). Selective serotonin reuptake inhibitors aggravate depression-associated dry eye via activating the NF-κb pathway. Invest Ophthalmol Vis Sci.

[92] Becker C, Jick SS, Meier CR (2017). Selective serotonin reuptake inhibitors and cataract risk: a case-control analysis. Ophthalmology.

[93] Klein BE, Klein R, Lee KE, Danforth LG (2001). Drug use and five-year incidence of age-related cataracts: the Beaver Dam Eye Study. Ophthalmology.

[94] Szabadi E, Gaszner P, Bradshaw CM (1981). Interaction of desipramine and amitriptyline with adrenergic mechanisms in the human iris in vivo. Eur J Clin Pharmacol.

[95] Wu A, Khawaja AP, Pasquale LR, Stein JD (2020). A review of systemic medications that may modulate the risk of glaucoma. Eye (Lond).

[96] Shifera AS, Leoncavallo A, Sherwood M (2014). Probable association of an attack of bilateral acute angle-closure glaucoma with duloxetine. Ann Pharmacother.

[97] Zheng W, Dryja TP, Wei Z, Song D, Tian H, Kahler KH, Khawaja AP (2018). Systemic medication associations with presumed advanced or uncontrolled primary open-angle glaucoma. Ophthalmology.

[98] Asherov J, Schoenberg A, Weinberger A (1982). Diplopia following ibuprofen administration. JAMA.

[99] Gamulescu MA, Schalke B, Schuierer G, Gabel VP (2006). Optic neuritis with visual field defect--possible ibuprofen-related toxicity. Ann Pharmacother.

[100] Alarfaj MA, Almater AI (2021). Olanzapine-induced acute angle closure. Am J Case Rep.

[101] Yogi TN, Bhusal A, Limbu S, B C P, Labh S, Kafle R (2023). Olanzapine-induced oculogyric crisis in a patient with mania without psychotic symptoms: a case report. Ann Med Surg (Lond).

[102] Nowrouzi A, Kafiabasabadi S, Rodriguez-Calzadilla M, Benitez-Del-Castillo J, Soto-Guerrero A, Diaz-Ramos A, Marques-Cavalcante KV (2021). Central retinal vein occlusion in a patient using the antipsychotic drug olanzapine: a case report. J Med Case Rep.

[103] Liperoti R, Pedone C, Lapane KL, Mor V, Bernabei R, Gambassi G (2005). Venous thromboembolism among elderly patients treated with atypical and conventional antipsychotic agents. Arch Intern Med.

[104] Zink M, Kuwilsky A, Knopf U (2007). Olanzapine-associated bilateral eyelid edema. J Clin Psychopharmacol.

[105] Hong H, Lyu IJ (2019). A case of skew deviation and downbeat nystagmus induced by lithium. BMC Ophthalmol.

[106] Dibajnia P, Mohammadinia M, Moghadasin M, Amiri MA (2012). Tear film break-up time in bipolar disorder. Iran J Psychiatry.

[107] Shah GK, Auerbach DB, Augsburger JJ, Savino PJ (1998). Acute thioridazine retinopathy. Arch Ophthalmol.

[108] Webber SK, Domniz Y, Sutton GL, Rogers CM, Lawless MA (2001). Corneal deposition after high-dose chlorpromazine hydrochloride therapy. Cornea.

[109] Srinivasan K, Gopalakrishna M (2020). Chlorpromazine-induced lenticular opacity. Indian J Pharmacol.

[110] Grenier E, Kirby J (1998). Chlorpromazine: case report and review of its ocular side-effects. Clin Eye Vision Care.

[111] Purhonen M, Koponen H, Tiihonen J, Tanskanen A (2012). Outcome of patients after market withdrawal of thioridazine: a retrospective analysis in a nationwide cohort. Pharmacoepidemiol Drug Saf.

[112] Evans PM, Lynch GL, Labelle P (2012). Effects of oral administration of diphenhydramine on pupil diameter, intraocular pressure, tear production, tear film quality, conjunctival goblet cell density, and corneal sensitivity of clinically normal adult dogs. Am J Vet Res.

[113] Foutch BK, Sandberg KA, Bennett ES, Naeger LL (2020). Effects of oral antihistamines on tear volume, tear stability, and intraocular pressure. Vision (Basel).

[114] Wren VQ (2000). Ocular and visual side effects of systemic drugs: clinically relevant toxicology and patient management. J Behav Optom.

[115] Lamberg O, Strome A, Jones F, Mleczek J, Jarocki A, Troost JP, Helfrich Y (2023). Ocular side effects of systemic isotretinoin - a systematic review and summary of case reports. J Dermatolog Treat.

[116] Irkec C, Eruyar E (2021). Ocular pseudo-myasthenic reaction during treatment with isotretinoin. Rev Neurol (Paris).

[117] Elubous KA, Toubasi AA, Elubous A, Alryalat SA, Abous H (2022). Ocular manifestations of systemic isotretinoin in patients with acne: a systemic review and meta-analysis. Cutan Ocul Toxicol.

[118] Chang DF, Tan JJ, Tripodis Y (2011). Risk factors for steroid response among cataract patients. J Cataract Refract Surg.

[119] Jobling AI, Augusteyn RC (2002). What causes steroid cataracts? A review of steroid-induced posterior subcapsular cataracts. Clin Exp Optom.

[120] Sutyawan WE, Widiastuti IA, Ryalino C (2019). Steroid induced cataract in Langerhans cell histiocytosis patient. Open Access Maced J Med Sci.

[121] Srikantia N, Mukesh S, Krishnaswamy M (2010). Crystalline maculopathy: a rare complication of tamoxifen therapy. J Cancer Res Ther.

[122] Gluvic Z, Obradovic M, Stewart AJ (2021). Levothyroxine treatment and the risk of cardiac arrhythmias - focus on the patient submitted to thyroid surgery. Front Endocrinol (Lausanne).

[123] Datta SG, S L SR, Dhananjaya MS, Tamminedi N, Nayak V, Kodapala S, Sarathi V (2023). Idiopathic intracranial hypertension following levothyroxine replacement therapy: systematic review and a case report. Indian J Endocrinol Metab.

[124] Strickler C, Pilon AF (2007). Presumed levothyroxine-induced pseudotumor cerebri in a pediatric patient being treated for congenital hypothyroidism. Clin Ophthalmol.

[125] Nickel JC, Moldwin R (2018). FDA BRUDAC 2018 criteria for interstitial cystitis/bladder pain syndrome clinical trials: future direction for research. J Urol.

[126] Vora RA, Patel AP, Melles R (2020). Prevalence of maculopathy associated with long-term pentosan polysulfate therapy. Ophthalmology.

[127] Hanif AM, Armenti ST, Taylor SC (2019). Phenotypic spectrum of pentosan polysulfate sodium-associated maculopathy: a multicenter study. JAMA Ophthalmol.

[128] Doiron RC, Bona M, Nickel JC (2020). Possible drug-induced, vision-threatening maculopathy secondary to chronic pentosan polysulfate sodium (Elmiron(®)) exposure. Can Urol Assoc J.

[129] Karuppannasamy D, Raghuram A, Sundar D (2014). Linezolid-induced optic neuropathy. Indian J Ophthalmol.

[130] Rao LV, Bhandary SV, Devi AR, Ninan A, Jain V, Veluri H (2006). Ocular toxicity of anti-tuberculous treatment. Kerala J Ophthalmol.

[131] Orssaud C, Nguyen DT, Rouzaud C (2022). [Screening and prevention of toxic optic neuropathies due to anti-mycobacterium therapies: proposal of recommendations]. J Fr Ophtalmol.

[132] Rodríguez-Marco NA, Solanas-Alava S, Ascaso FJ, Martínez-Martínez L, Rubio-Obanos MT, Andonegui-Navarro J (2014). [Severe and reversible optic neuropathy by ethambutol and isoniazid]. An Sist Sanit Navar.

[133] Fraunfelder FW, Fraunfelder FT, Jensvold B (2003). Scleritis and other ocular side effects associated with pamidronate disodium. Am J Ophthalmol.

[134] Fraunfelder FW, Winthrop K, Suhler E, Choi D, Fraunfelder FT (2009). Postmarketing surveillance rates of uveitis and scleritis with bisphosphonates among a national veteran cohort. Retina.

